# The influence of few-layer graphene on the gas permeability of the high-free-volume polymer PIM-1

**DOI:** 10.1098/rsta.2015.0031

**Published:** 2016-02-13

**Authors:** Khalid Althumayri, Wayne J. Harrison, Yuyoung Shin, John M. Gardiner, Cinzia Casiraghi, Peter M. Budd, Paola Bernardo, Gabriele Clarizia, Johannes C. Jansen

**Affiliations:** 1 School of Chemistry, University of Manchester, Manchester M13 9PL, UK; 2 Manchester Institute of Biotechnology and School of Chemistry, University of Manchester, Manchester M1 7DN, UK; 3 Institute on Membrane Technology (ITM-CNR), Via P. Bucci, cubo 17/C, Rende (CS) 87036, Italy

**Keywords:** mixed matrix membranes, polymers of intrinsic microporosity, graphene, gas permeation

## Abstract

Gas permeability data are presented for mixed matrix membranes (MMMs) of few-layer graphene in the polymer of intrinsic microporosity PIM-1, and the results compared with previously reported data for two other nanofillers in PIM-1: multiwalled carbon nanotubes functionalized with poly(ethylene glycol) (f-MWCNTs) and fused silica. For few-layer graphene, a significant enhancement in permeability is observed at very low graphene content (0.05 vol.%), which may be attributed to the effect of the nanofiller on the packing of the polymer chains. At higher graphene content permeability decreases, as expected for the addition of an impermeable filler. Other nanofillers, reported in the literature, also give rise to enhancements in permeability, but at substantially higher loadings, the highest measured permeabilities being at 1 vol.% for f-MWCNTs and 24 vol.% for fused silica. These results are consistent with the hypothesis that packing of the polymer chains is influenced by the curvature of the nanofiller surface at the nanoscale, with an increasingly pronounced effect on moving from a more-or-less spherical nanoparticle morphology (fused silica) to a cylindrical morphology (f-MWCNT) to a planar morphology (graphene). While the permeability of a high-free-volume polymer such as PIM-1 decreases over time through physical ageing, for the PIM-1/graphene MMMs a significant permeability enhancement was retained after eight months storage.

## 1. Introduction

**Figure 1.**
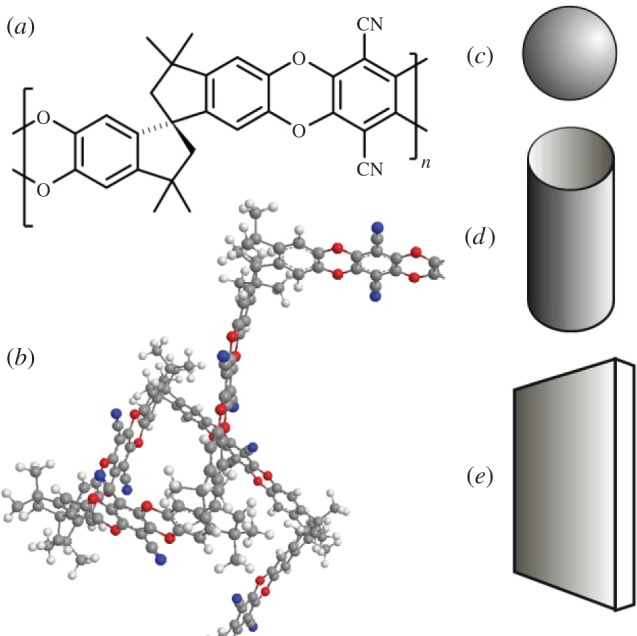
(*a*) Chemical structure of the polymer of intrinsic microporosity PIM-1. (*b*) Molecular model of a fragment of PIM-1 showing its contorted structure. Idealized nanofiller morphologies (*c*) spherical, (*d*) cylindrical and (*e*) planar. (Online version in colour.)

Membranes are currently used for a variety of industrial gas separations, including nitrogen generation from air and the removal of CO_2_ from natural gas [1]. However, for many potential large-scale applications, such as the capture of CO_2_ from power station flue gases, there is a need for new materials that offer high permeability combined with good selectivity. High-free-volume polymers, such as polymers of intrinsic microporosity (PIMs), have attracted attention for their high gas permeabilities [2,3]. However, they are susceptible to physical ageing, which leads to a reduction in permeability over time [4,5]. The addition of an inorganic, metal-organic or organic filler to a polymer, to form a mixed matrix membrane (MMM) [6], can give synergistic improvements in the permeation properties, help to control ageing effects, and enhance the mechanical performance. Here, the effect of graphene on the gas permeability of PIM-1 (figure 1*a*) [7–9], the archetypal solution-processable PIM, is compared with data of Khan *et al*. [10,11] for multiwalled carbon nanotoubes functionalized with poly(ethylene glycol) (f-MWCNTs) and data of Ahn *et al.* [12] for a fused silica nanofiller.

Simple models of MMMs predict that the addition of an impermeable nanofiller to a permeable matrix will reduce permeability compared with the unfilled material. For example, the Maxwell model [13] predicts that the permeability *P*_MMM_ of a MMM containing a volume fraction *ϕ*_F_ of impermeable, spherical filler particles within a polymer of permeability *P*_P_, is given by equation (??).
1.1
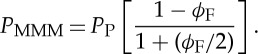
Pinnau & He [14] reported that the addition of non-porous fillers to glassy polymers can actually increase permeability. This counter-intuitive effect has been confirmed for non-porous inorganic fillers, such as fumed silica [12], MgO [15] and TiO_2_ [16], in various polymers.

The permeability of a glassy polymer is linked to the amount and distribution of free volume within the material. Glasses are in a non-equilibrium state, and many factors may influence the way the molecules pack together and, thus, the free-volume distribution. High-free-volume glassy polymers have high permeabilities as a result of frustrated packing of the constituent macromolecules. In a PIM, this is a consequence of the molecular structure of the polymer chain, which is composed of fused-ring sequences interrupted by sites of contortion such as spiro-centres (figure 1*b*). The randomly twisted shape makes it difficult for molecules to pack, and because there are no single bonds in the backbone about which rotation can occur, they cannot undergo the types of conformational change that enable conventional polymers to rearrange and fill space.

The presence of a nanofiller surface restricts the conformational freedom of polymer chains in its vicinity, which may further frustrate the ability of the chains to pack together, thus increasing free volume and enhancing permeability. It is a reasonable hypothesis that the effect of a nanofiller will depend not only on its available surface area but also on the curvature of the surface at the nanoscale. Here we compare an essentially planar filler (graphene), with a cylindrical nanofiller (f-MWCNTs), and a filler composed of more-or-less spherical nanoparticles (fumed silica) (figure 1*c*–*e*).

The term ‘graphene’ may be applied to a wide range of materials. A classification framework for graphene-based materials has been suggested [17] and a recommended nomenclature proposed [18]. The graphene family includes graphene itself (a single-atom-thick sheet), bilayer graphene, few-layer graphene, etc. Material with more than *ca* 10 regularly stacked layers is generally regarded as graphitic. For the present work, few-layer graphene was considered a suitable planar nanofiller for comparison with multiwalled carbon nanotubes.

## 2. Experimental

### (a) Materials

Tetrafluoroterephthalonitrile (TFTPN, 98%, Aldrich) was purified by sublimation; it was heated to around 150°C and the pure product collected without vacuum. 5,5^′^,6,6^′^-Tetrahydroxy-3,3,3^′^,3^′^-tetramethyl-1,1^′^-spirobisindane (TTSBI, 98%, Alfa Aesar) was dissolved in methanol and re-precipitated from dichloromethane before use. Anhydrous K_2_CO_3_ (99.0%, Fisher) was dried in an oven at 110°C overnight before use. Anhydrous dimethylacetamide (DMAc), toluene and methanol (MeOH), were purchased from Sigma-Aldrich and used as received. Natural graphite was purchased from NGS Naturgraphite GmbH.

### (b) Methods

Gel permeation chromatography (GPC) measurements were carried out using a Viscotek GPC max VE 2001 instrument with two PL mixed B columns and a refractive index detector. Tetrahydrofuran was used as solvent at a flow rate of 1 cm^3^ min^−1^ and the injection volume was 100 μl. Polystyrene standards of known molar mass were used for calibration.

^1^H nuclear magnetic resonance (NMR) spectroscopy was carried out using a Bruker 400 MHz spectrometer. For NMR sample preparation, PIM-1 (≈5 mg) was dissolved in deuterochloroform (CDCl_3_, Aldrich, 99.8% atom D) and transferred into a 5 mm NMR tube.

Ultraviolet–visible (UV–Vis) spectroscopy was carried out using a Cary 60 UV–Vis spectrophotometer at room temperature.

### (c) Synthesis of PIM-1

PIM-1 was synthesized by a method based on that of Du *et al*. [19]. TFTPN (2.001 g, 0.01 mol), TTSBI (3.45 g, 0.01 mol), anhydrous K_2_CO_3_ (0.03 mol), DMAc (20 ml) and toluene (10 ml) were added to a round-bottomed flask equipped with a mechanical stirrer, nitrogen inlet and Dean–Stark trap. The mixture was refluxed at 160°C for 40 min, then the viscous solution was added to methanol to precipitate the product. The yellow product was dissolved in chloroform and reprecipitated from methanol. Further purification was carried out by refluxing the precipitate in deionized water overnight, then it was dried overnight in a vacuum oven at 110°C. GPC: *M*_n_=43000, *M*_w_=170000, *M*_w_/*M*_n_=4.0. ^1^H NMR (400 MHz; CDCl_3_): *δ*6.81 (br, s, 2H), 6.42 (br, s, 2H), 2.33 (br, s, 2H), 2.17 (br, s, 2H), 1.36 (br, s, 6H), 1.31 (br, s, 6H). IR (ATR; cm^−1^): 3000–2800, 2238, 1443, 1262, 1107, 1009, 751. Elemental analysis, calculated for C_29_H_20_N_2_O_4_ (wt.%): C, 75.64; H, 4.38; N, 6.08. Found: C, 71.99; H, 4.17; N, 6.02.

### (d) Preparation of PIM-1/graphene solutions and membrane casting

A stock PIM-1/graphene dispersion was prepared as follows: natural graphite (600 mg) and PIM-1 (1.4 g) were added to chloroform (100 ml). The solution was sonicated for 84 h (Elmasonic P 70H, 220 W effective ultrasonic power, 37 kHz ultrasonic frequency) and then centrifuged twice at 6000 r.p.m. for 20 min to remove graphitic particles. Although prolonged sonication of a polymer solution might be expected to cause cleavage of polymer chains, control experiments with PIM-1 solutions showed little effect on number-average molar mass, *M*_*n*_, and only a modest decrease in weight-average molar mass, *M*_*w*_. To form the membrane with the highest graphene content (sample KA1-7-12(3)), a 5 ml portion of the stock solution was transferred into a 7.8 cm diameter flat-bottomed glass petri dish, and the solvent was allowed to evaporate over 3 days. For preparation of membranes with lower graphene contents, portions of the stock PIM-1/graphene solution were mixed with portions of a pure PIM-1 solution of concentration 35 mg ml^−1^. For samples KA-1-7(1), KA-1-7(2) and KA-1-7(3), 2 ml of stock solution were mixed with 2, 5 and 10 ml, respectively, of PIM-1 solution. For samples KA-1-7(5) and KA-1-7(6), 4 ml of stock solution were mixed with 20 and 40 ml, respectively, of PIM-1 solution. A 7.8 cm petri dish was used for samples KA-1-7(1) and KA-1-7(6), and a 6.8 cm dish for other samples.

### (e) Determination of graphene content

For determination of the graphene content, membrane KA-1-7(5) (0.23 g) was redissolved in 23 ml of chloroform. The graphene concentration of the solution, *C*_G_, was determined by UV–Vis spectroscopy. The absorption was measured at 660 nm and *C*_G_ evaluated using a value of *α*=3620 ml mg^−1^ m^−1^ for the absorption coefficient, as determined by the Coleman group [20,21]. This gave a graphene weight fraction of 0.0018. Hence, it was determined that the stock PIM-1/graphene solution contained 0.349 mg ml^−1^ graphene, as well as 14 mg ml^−1^ PIM-1. The graphene contents of membranes used for permeation measurements were then calculated from the known dilutions. For calculation of the percentage graphene by volume, the graphene was assumed to have the same density as graphite (2.2 g cm^−3^).

### (f) Gas permeation measurements

Single gas permeation measurements were carried out in a fixed volume/pressure increase apparatus (GKSS, Germany) at 25°C at a feed pressure of 1 bar, as described previously [22,23].

## 3. Results and discussion

### (a) PIM-1/graphene MMMs

Self-supported PIM-1/graphene MMMs were obtained by slow evaporation of the solvent from PIM-1/graphene dispersions in chloroform. A stock PIM-1/graphene dispersion was prepared by ultrasound-assisted exfoliation of natural graphite into a PIM-1 solution. Dilution of the stock dispersion with fresh PIM-1 solution enabled dispersions of various concentrations to be obtained, and hence MMMs with various graphene contents as indicated in table 1. While chloroform alone is not a particularly good solvent for graphene exfoliation, the PIM-1 acts to stabilize the dispersion through interactions with the graphene [24]. Raman spectroscopy [25–27] of flakes in a PIM-1/graphene MMM shows restacked few-layer graphene, and excludes the presence of species with graphitic AB stacking (see the electronic supplementary material).

**Table 1.** Graphene content, thickness and gas permeabilities of PIM-1 and PIM-1/graphene membranes after methanol-treatment and after ageing for *ca* eight months.

	graphene content				permeability (barrer)					
membrane	(wt/%)	(vol.%)	(days)	(μm)	CO_2_	H_2_	He	O_2_	CH_4_	N_2_
PIM-1	0	0	0	59	5120	3210	1610	1130	340	270
KA1-7(6)	0.00096	0.046	0	352	12 700	4660	1770	2260	1450	870
KA1-7(3)	0.0018	0.088	0	100	9840	4730	1890	1850	800	570
KA1-7(2)	0.0034	0.164	0	52	7830	4470	1830	1560	550	410
KA1-7(1)	0.0071	0.338	0	24	3410	3860	1950	820	160	170
KA1-7-12(3)	0.0243	0.338	0	86	5150	3210	1390	1040	390	270
PIM-1	0	0	244	57	3670	2720	1220	730	200	160
KA1-7(6)	0.00096	0.046	226	351	9240	3970	1550	1800	980	620
KA1-7(3)	0.0018	0.088	236	94	6660	3450	1420	1250	460	340
KA1-7(2)	0.0034	0.164	236	50	5680	3210	1350	1,070	330	260

Single gas permeabilities for PIM-1/graphene MMMs, and for the batch of PIM-1 used in this work, are included in table 1. The measurements are for membranes which have been soaked in methanol then dried. The methanol-treatment is used to remove residual solvents and to reverse effects of the previous membrane history. Single gas permeabilities decrease in the sequence CO_2_ > H_2_ > He ≈ O_2_ > CH_4_ > N_2_. In the simplest model of membrane permeation, the solution-diffusion model, permeability coefficient *P* is the product of a diffusion coefficient *D* and a solubility or sorption coefficient *S* (equation (3.1)).
3.1
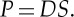
The permeability sequence CO_2_ > H_2_ is the reverse of that observed for the majority of glassy polymers, and may be attributed to strong sorption enhancing CO_2_ permeability. It should be noted, however, that if the sorption term is too strong, diffusion may be restricted and the permeability decreased, as has been observed for amine-modified PIM-1 [23].

A significant enhancement in gas permeability is observed at low graphene content. This is discussed further and compared with the effect of other nanofillers below.

### (b) Comparison of nanofillers

**Figure 2.**
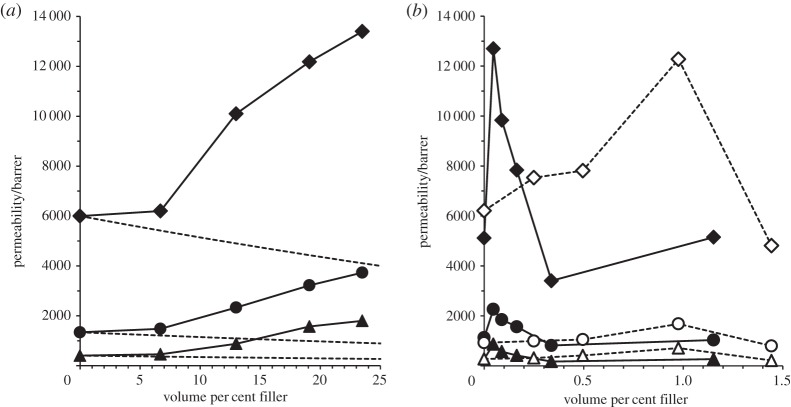
Dependence of CO_2_ (diamonds), O_2_ (circles) and N_2_ (triangles) permeability on nanofiller loading for MMMs of PIM-1 with (*a*) fused silica [12] and (*b*) graphene (solid symbols) (this work) and f-MWCNTs (open symbols) [10]. The dashed lines in (*a*) indicate the prediction of the Maxwell model for an impermeable filler.

Data of Ahn *et al*. [12] for methanol-treated MMMs of hydrophobic fumed silica (Cabosil TS 530) in PIM-1 are compared with the Maxwell model (equation (??)) in figure 2*a*. It can be clearly seen that while the Maxwell model predicts a decrease in gas permeability, the experimental results show a significant increase in the effective permeability of the MMM with increasing filler loading over the range 7–24 vol.%. Filler loadings above 24 vol.% were not investigated; it is difficult to achieve homogeneous dispersions at higher loadings. The nanoparticles used in that study were reported to have particle diameters in the range 11.1–13.3 nm, although there was evidence of larger aggregates in the MMMs. Filler density and Brunauer–Emmett–Teller (BET) surface area were reported as 2.2 g cm^−3^ and 205−245 m^2^ g^−1^, respectively.

Permeability data from Khan *et al.* [10] for methanol-treated PIM-1/f-MWCNT MMMs are shown in figure 2*b*. The MWCNTs used for functionalization (FutureCarbon GmbH) were reported to have 8–12 walls and diameter in the range 12–15 nm. The BET surface area was 250 m^2^ g^−1^, similar to the fused silica described above. For the plot in figure 2*b*, a density of 2.1 g cm^−3^ was assumed in order to calculate vol.% filler; in practice, the density may vary depending on the diameter and number of walls [28]. It can be seen that gas permeabilities increase up to *ca* 1 vol.% filler, and then decrease.

Permeability data for methanol-treated PIM-1/graphene MMMs from the present work are included in figure 2*b*. For calculation of vol.% filler, the density of bulk graphite (2.2 g cm^−3^) was assumed. A similar pattern is seen with graphene as for f-MWCNTs, with an initial increase followed by a decrease with increasing filler content. While PIM-1 itself can exhibit substantial differences in permeability depending on sample history, it is noteworthy that broadly similar results were obtained by three different laboratories working with three different fillers, the CO_2_ permeability for methanol-treated PIM-1 being in the range 5000–6200 barrer, and the highest CO_2_ permeability achieved for these MMMs being about double, in the range 12 200–13 400 barrer. The significant difference between the fillers is the filler loading at which the greatest permeability enhancement is seen, which increases from *ca* 0.05 vol.% for few-layer graphene, to *ca* 1 vol.% for f-MWCNTs, to *ca* 24 vol.% for fused silica.

The maximum in permeability observed with increasing content of graphene or f-MWCNTs, and which would presumably also be seen if it were possible to push fused silica loadings to higher levels, may be attributed to competition between an enhancement in permeability through disruption of polymer chain packing, and a reduction in permeability due to the added bulk of the filler, perhaps coupled with the effects of aggregation of the filler at higher concentration. The differences in nanofiller loadings at which the maximum occurs may in part reflect differences in accessible surface area. However, for the MWCNT and fused silica nanofillers the surface areas appear to be similar, but there is a large difference in the nanofiller content at which the permeability enhancement is observed, suggesting that the morphology of the nanofiller plays an important role. For a single graphene sheet the theoretical surface area is 2630 m^2^ g^−1^ (1315 m^2^ g^−1^ per side [29]), but it will be much less for few-layer graphene in the MMMs. It, therefore, seems likely that the sheet-like morphology is at least partly responsible for the very low graphene content (0.05 vol.%) at which permeability enhancement is observed. These results are consistent with the hypothesis that on moving from a spherical, to a cylindrical, to a planar nanofiller, pronounced effects on polymer chain packing, and thus on permeability, may be achieved at increasingly lower nanofiller contents.

### (c) Effect of ageing

**Figure 3.**
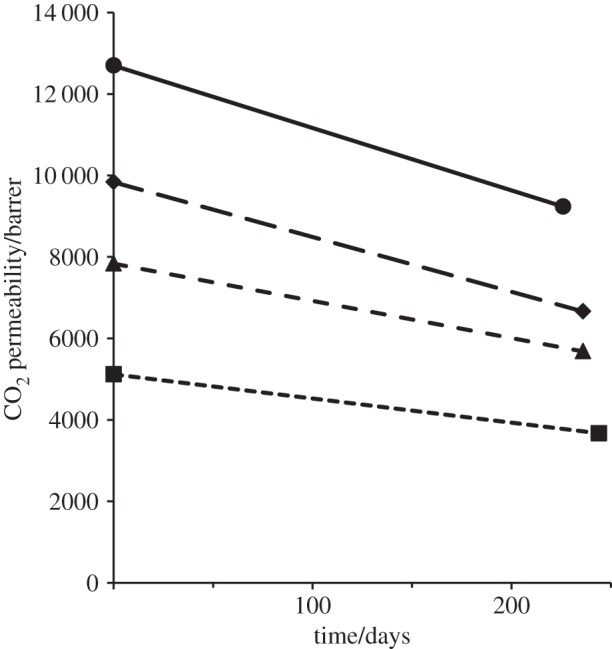
Effect of ageing for *ca* eight months on the CO_2_ permeability of PIM-1 (square) and PIM-1/graphene MMMs at nanofiller loadings of 0.046 vol.% (circle), 0.088 vol.% (diamond) and 0.164 vol.% (triangle). The lines are guides to the eye.

Khan *et al*. [11] observed that thin-film composite membranes with a PIM-1/f-MWCNT active layer on a porous polyacrylonitrile support showed better long-term stability than membranes prepared with PIM-1 alone as the active layer. In this work, the gas permeabilities of self-supported PIM-1/graphene membranes were re-measured after *ca* eight months storage under ambient conditions (table 1). The changes in CO_2_ permeability for different graphene loadings are illustrated in figure 3. While a loss of permeability is observed over time in all cases, a substantial permeability enhancement is retained after eight months for the PIM-1/graphene MMMs, as compared to neat PIM-1.

## 4. Conclusion

For this series of samples, the addition of just 0.05 vol.% few-layer graphene to PIM-1 gave a substantial enhancement in gas permeability; for CO_2_, it was more than twofold. Even after eight months of storage the permeability was substantially higher than that observed for the unfilled polymer. Other nanofillers exhibit similar permeability enhancements, but higher loadings are required. This is consistent with the hypothesis that packing of the polymer chains is influenced by the curvature of the nanofiller surface at the nanoscale, with an increasingly pronounced effect on moving from a more-or-less spherical nanoparticle morphology (fused silica) to a cylindrical morphology (f-MWCNT) to a planar morphology (graphene).

## Supplementary Material

Characterization of graphene in PIM-1 by Raman spectroscopy
