# Supplementary material for: The influence of few-layer graphene on the gas permeability of the high-free-volume polymer PIM-1
Source: Philos Trans A Math Phys Eng Sci. 2016 Feb 13;374(2060):20150031. doi: 10.1098/rsta.2015.0031 (PMC4696075; doi:10.1098/rsta.2015.0031)
Supplement: Characterization of graphene in PIM-1 by Raman spectroscopy [file rsta20150031supp1.docx]

**The influence of few-layer graphene on the gas permeability of the high-free-volume polymer PIM-1**

Khalid Althumayri, Wayne J. Harrison, Yuyoung Shin, John M. Gardiner, Cinzia Casiraghi,

Peter M. Budd, Paola Bernardo, Gabriele Clarizia and Johannes C. Jansen

*University of Manchester, UK, and Institute on Membrane Technology (ITM-CNR), Italy*

**Characterization of graphene in PIM-1 by Raman spectroscopy**

PIM-1/graphene dispersions were drop-cast on a silicon substrate. Individual flakes (~40) embedded in the polymer were examined by Raman spectroscopy, using a Renishaw Raman spectrometer, with laser wavelength of 633 nm, 1200 g/mm grating, and 50X objective. Low laser power (~ 1.0 mW on sample) was used to avoid damage.

Raman spectroscopy offers a simple and fast analysis of graphene sheet thickness with the evolution of the 2D peak shape. However, during exfoliation, adsorption of the solvent, edge effects and strain can strongly affect the Raman spectrum of Liquid-Phase Exfoliated (LPE) graphene, therefore it is more difficult to analyze this material, compared to mechanically exfoliated graphene. This makes the Raman analysis only qualitative.

The Raman spectrum of LPE graphene is always characterized by a D peak, which is typically activated by the edges of the flakes, being of size comparable to the laser spot. The 2D peak shape, routinely used to identify graphene, can show very complex line-shapes due to re-stacking of the flakes and other effects. However, we have shown [[1](#_ENREF_1), [2](#_ENREF_2)] that the shape of the 2D peak can still be used to distinguish between single-layer graphene (SLG), few-layer graphene (FLG) and graphite (>10 layers). Here, we used the same protocol, *i.e.* we look at the symmetry of the 2D mode, when fitting this peak with a Lorentzian lineshape. A very symmetric peak is attributed to SLG, while if the peak can be well fitted with two separate components, it is attributed to graphite; if the peak is strongly asymmetric, then it is attributed to FLG. This protocol has been shown to be in good agreement with results obtained by Transmission Electron Microscopy [[1](#_ENREF_1), [2](#_ENREF_2)].

**Supplementary Figure 1** shows a typical Raman spectrum of a flake deposited from a dispersion prepared by exfoliation of graphite (initial concentration 6 mg mL^-1^) in a solution of PIM-1 in chloroform (14 mg mL^-1^), sonicated for 84 h and then centrifuged at 10,000 rpm for 20 min. The graphene concentration in the final dispersion was determined by UV-Vis spectroscopy as 0.132 mg mL^-1^. The first order Raman spectrum of PIM-1 shows peaks that overlap with the graphene D and G peaks. Fortunately, the 2D peak is lying (at 633 nm excitation wavelength) in a region not obscured by the PIM-1 Raman modes, so if the concentration of graphene-based material in the mixed matrix membrane (MMM) is high enough, it is then possible clearly to observe the 2D peak. A typical 2D peak observed in a graphene/PIM-1 MMM is asymmetric (**Supplementary Figure 2**) and does not correspond to any AB-stacked graphitic material (either thin or thick) [[3](#_ENREF_3)]. This can be interpreted as re-stacked few-layer graphene (*i.e*., graphene that has partially reassembled, but without the regularity of the AB stacking found in natural graphite).

**References**

1. Haar S, Ciesielski A, Clough J, Yang H, Mazzaro R, Richard F, Conti S, Merstorf N, Cecchini M, Morandi V, Casiraghi C, Samori P. 2015 A Supramolecular Strategy to Leverage the Liquid-Phase Exfoliation of Graphene in the Presence of Surfactants: Unraveling the Role of the Length of Fatty Acids. *Small*. **11**, 1691-1702. (doi: 10.1002/smll.201402745)

2. Ciesielski A, Haar S, El Gemayel M, Yang H, Clough J, Melinte G, Gobbi M, Orgiu E, Nardi MV, Ligorio G, Palermo V, Koch N, Ersen O, Casiraghi C, Samori P. 2014 Harnessing the Liquid-Phase Exfoliation of Graphene Using Aliphatic Compounds: A Supramolecular Approach. *Angew. Chem., Int. Edn*. **53**, 10355-10361. (doi: 10.1002/anie.201402696)

3. Ferrari AC, Meyer JC, Scardaci V, Casiraghi C, Lazzeri M, Mauri F, Piscanec S, Jiang D, Novoselov KS, Roth S, Geim AK. 2006 Raman Spectrum of Graphene and Graphene Layers. *Phys. Rev. Lett*. **97**, 187401/187401-187401/187404. (doi: 10.1103/PhysRevLett.97.187401)

**Supplementary Figure 1:** Raman spectrum of PIM-1 (top) compared with a typical Raman spectrum of a flake in a graphene/PIM-1 MMM containing a high concentration of graphene-based material (bottom). While the G and D peak are difficult to distinguish from the Raman features of PIM-1, the 2D peak of graphene, measured at 633 nm, is well visible, allowing for qualitative thickness analysis.

**Supplementary Figure 2:** Lorentzian fitting of the 2D peak of a graphene flake in PIM-1, showing that the peak is slightly asymmetric. This can be interpreted as re-stacked few-layer graphene (*i.e*., graphene that has partially re-assembled, but without the regularity of the AB stacking found in natural graphite).
